# Systematic review of the effectiveness of health promotion interventions targeting obesity prevention in school-based staff

**DOI:** 10.1093/heapro/daac061

**Published:** 2022-07-05

**Authors:** Amy Hill, Laura Alston, Cindy Needham, Anna Peeters, Anthony D LaMontagne, Melanie Nichols

**Affiliations:** Global Obesity Centre (GLOBE), Institute for Health Transformation, Faculty of Health, Deakin University, Geelong 3220, VIC, Australia; Global Obesity Centre (GLOBE), Institute for Health Transformation, Faculty of Health, Deakin University, Geelong 3220, VIC, Australia; Deakin Rural Health, Faculty of Health, Deakin University, Geelong 3220, VIC, Australia; Colac Area Health, Research, Colac 3250, VIC, Australia; Global Obesity Centre (GLOBE), Institute for Health Transformation, Faculty of Health, Deakin University, Geelong 3220, VIC, Australia; Institute for Health Transformation, Faculty of Health, Deakin University, Geelong 3220, VIC, Australia; Institute for Health Transformation, Faculty of Health, Deakin University, Geelong 3220, VIC, Australia; Global Obesity Centre (GLOBE), Institute for Health Transformation, Faculty of Health, Deakin University, Geelong 3220, VIC, Australia

**Keywords:** nutrition, physical activity, health promotion, obesity prevention, occupational health

## Abstract

School-based employee interventions can benefit the health of staff and have the potential to influence the health of school students through role-modelling. However, interventions within schools typically focus on students, with very few studies addressing obesity and related health behaviours among school staff. A systematic review of the peer-reviewed literature published between January 2000 and May 2020 was undertaken to synthesize the evidence on the impact that school-based obesity prevention programmes have on the staff they employ. Search terms were derived from four major topics: (i) school; (ii) staff; (iii) health promotion and (iv) obesity. Terms were adapted for six databases and three independent researchers screened results. Studies were included if they reported on the outcomes of body weight, dietary behaviours and/or physical activity. Of 3483 papers identified in the search, 13 studies met the inclusion criteria. All 13 studies included an intervention that focussed on improving nutrition, physical activity or both. All included studies demonstrated a positive outcome for either dietary intake, weight or body mass index or physical activity outcomes, however not all results were statistically significant. The included studies showed promising, although limited, impacts on employee health outcomes. This review demonstrated a lack of global focus and investment in interventions targeting school staff, particularly in contrast to the large amount of research on school-based health promotion initiatives focussed on students. There is a need for further research to understand effective interventions to promote health and prevent obesity in this large, diverse and influential workforce.

## INTRODUCTION

The Global Burden of Disease Study estimated that obesity affected 2 billion people globally in 2015, imposing an economic burden of US $2.0 trillion in health care costs, mortality and permanent disability, slowed economic growth and lost productivity (Tremmel *et al.*, 2017; Swinburn *et al.*, 2019). If current trends continue, it is estimated that more than half of the world’s population will meet the classifications of overweight and obesity by 2030, posing further significant risks to human health (Kelly *et al.*, 2008). High quality evidence is needed to understand ways to address adult overweight and obesity that can deliver sustained population level benefits.

Workplaces are an important setting for health promotion, given that many adults spend a large proportion of their week in employment settings, and that poor health may lead to detrimental impacts not only for employees themselves, but also for productivity and organizational success (Chalupka, 2011). Workplace health promotion interventions have been shown to be effective in relation to obesity prevention in various employment sectors, including health care settings, office-based workplaces and university settings (Anderson *et al.*, 2009; Verweij *et al.*, 2011; Power *et al.*, 2014; Weerasekara *et al.*, 2016; Tam and Yeung, 2018; Proper and van Oostrom, 2019). A recent review of reviews on the effectiveness of workplace health promotion interventions that synthesized evidence from 23 reviews, found strong evidence for favourable impacts on weight-related outcomes and prevention of mental health disorders (Proper and van Oostrom, 2019).

Despite published evidence on the effectiveness of health promotion interventions in various workplaces, little is known about their effectiveness when targeting school staff and the unique school workplace environment that is characterized by high job demands, busy workload and inflexibility of staff time (Katz *et al.*, 2005).

Health promotion interventions focussed on the education sector have become of increasing interest for governments and policy makers given not only the potential for school employees’ own health benefits, but also the potential for school employees to be positive role-models of healthful behaviour for the children they teach and interact with (Hartline-Grafton *et al.*, 2009; Snelling *et al.*, 2013; Herbert *et al.*, 2017). The World Health Organization’s Health Promoting Schools Framework further highlights the opportunities available through schools towards taking a whole of setting approach to obesity prevention (World Health Organisation, 2017). A recent review of the framework demonstrated its effectiveness with regard to some weight-related and behavioural (diet and physical activity) contributors to obesity in children (Langford *et al.*, 2014). However, the majority of studies only targeted school students, with little published evidence available on the effectiveness of obesity-related health promotion interventions for school staff (Katz *et al.*, 2005).

The Australian school-based workforce comprises a diverse group of adults with a broad range of ages and ethnicities across all localities nationally (Department of Education, 2014). The school-based workforce includes the categories of teaching staff (approximately 70% of the Australian school-based workforce), and non-teaching staff comprising specialist support staff, administrative and clerical staff (including teacher aides and assistants), building operations, general maintenance and other staff. In 2019, there were over 288 000 full-time equivalent (FTE) teaching staff and 132 000 FTE non-teaching staff employed in Australian schools, with over 3.9 million enrolled students (Australian Bureau of Statistics, 2019).

A recent Australian study explored potential opportunities for schools to support healthy eating and physical activity amongst staff (Huse *et al.*, 2020) and found that key barriers to school staff eating healthily and being physically active included lack of time and support for these behaviours and lack of necessary physical infrastructure in the workplace (Huse *et al.*, 2020). Recommendations from this study included adopting a whole of school health promotion policy to support teachers to pursue healthier lifestyles and reduce workplace stress (Huse *et al.*, 2020).

Given the potential that school-based interventions have for improving health, evidence synthesis with a specific focus on staff members health and wellbeing is needed in order to inform future interventions, health promotion programmes and research. We therefore sought to:

1. Synthesize the literature from quantitative studies reporting on the effectiveness of obesity prevention interventions for school staff; and
2. Identify the common characteristics of successful interventions.

## METHODS

### Eligibility criteria

Studies were included if (i) they were published in English between 2000 and 2020; (ii) intervention participants were school staff (primary or secondary schools or equivalent), including teaching, administrative and leadership staff; (iii) the study included a health promotion intervention; (iv) the study design included a control group or before and after measures [i.e. randomized controlled trials (RCTs), quasi-experimental and non-randomized designs] and (v) outcome measures included at least one of; a measure of weight [including body mass index (BMI)], dietary behaviours and/or physical activity. Literature reviews, conference abstracts and editorials were excluded. The decision to include studies from the year 2000 onwards was made following initial trials of the search strategy where it was found that the majority of school-based health promotion literature was published after this date.

### Search strategy

The search strategy was developed following a preliminary review of workplace and school-based health promotion interventions. The search was initially conducted on 25 March 2019 and updated on the 14 May 2020 to capture any new publications since initial search. The following databases were included in the search: Medline, CINAHL, EMBASE, Health and Society database (Informit), ERIC and Google Scholar; the search strategy was adjusted for each database and limited to original research published in peer-reviewed journals, published in the English language. The search strategy terms used and journal-specific syntax can be found in Supplementary Table 1. Search terms included combinations, truncations and synonyms of the following: (i) ‘School OR Staff*’ OR teacher* OR employee* OR worker* OR workplace, (ii) Intervention* OR program* OR ‘health promotion’ OR prevent* OR strategy* OR initiative*, (iii) Diet* OR nutrition* OR eating OR consumption OR weight OR overweight OR obesity* OR ‘physical* activity’ OR ‘sedentary behaviour’ OR exercise. Additional articles were identified via a manual search of reference lists.

### Study selection

Search results were extracted from databases into Endnote X9 (Clarivate Analytics, USA). Results were then loaded into systematic review software Covidence (Covidence systematic review software, Veritas Health Innovation, Melbourne, Australia), and three reviewers independently screened titles and abstracts using a pre-determined eligibility assessment form. Full texts of all articles included after title and abstract screening were also each reviewed by two reviewers. Any discrepancies through the review process were adjudicated by a senior researcher and resolved by consensus discussion.

### Data extraction

Data extraction was completed by the lead researcher; with data from a 10% subsample (*n* = 3) of papers extracted by a second reviewer which were checked for agreement which was achieved. Key study characteristics were identified and extracted into a pre-determined data extraction form to enable identification of prominent and recurrent themes. The following data were extracted: author, year of publication; type of school (i.e. primary/elementary, secondary); country; study design; participants (i.e. teachers, administration staff); intervention strategies; intervention timeframe; outcome measures; results; conclusions. The detailed data extraction results can be found in Supplementary Table 2.

### Data synthesis and analysis

The key characteristics of the included studies were summarized from the information collected in the data extraction form. The primary outcomes of interest were statistically significant changes in diet, physical activity or unhealthy weight measures among school staff as a result of the school-based intervention. Where possible, for all study arms, the mean or median of primary outcome measures was recorded at baseline, post-intervention and any additional follow-up(s). Measures of error were standard error or SD and associated *p* values for change between or within groups at follow-up(s), and over time were recorded if available. The diverse characteristics of included studies precluded a meta-analysis, with variation in study design, outcome measures and intervention strategies.

### Quality assessment

Following data extraction, assessment of methodological quality of included studies was undertaken using the Quality Assessment Tool for Before-After (Pre-Post) Studies with No Control Group (National Heart Lung and Blood Institute, 2020). Each study was assigned an overall quality rating; good, fair or poor, based on an evaluation of risk of bias through key concepts raised in the 12 questions of the quality assessment tool.

## RESULTS

**Fig. 1:**
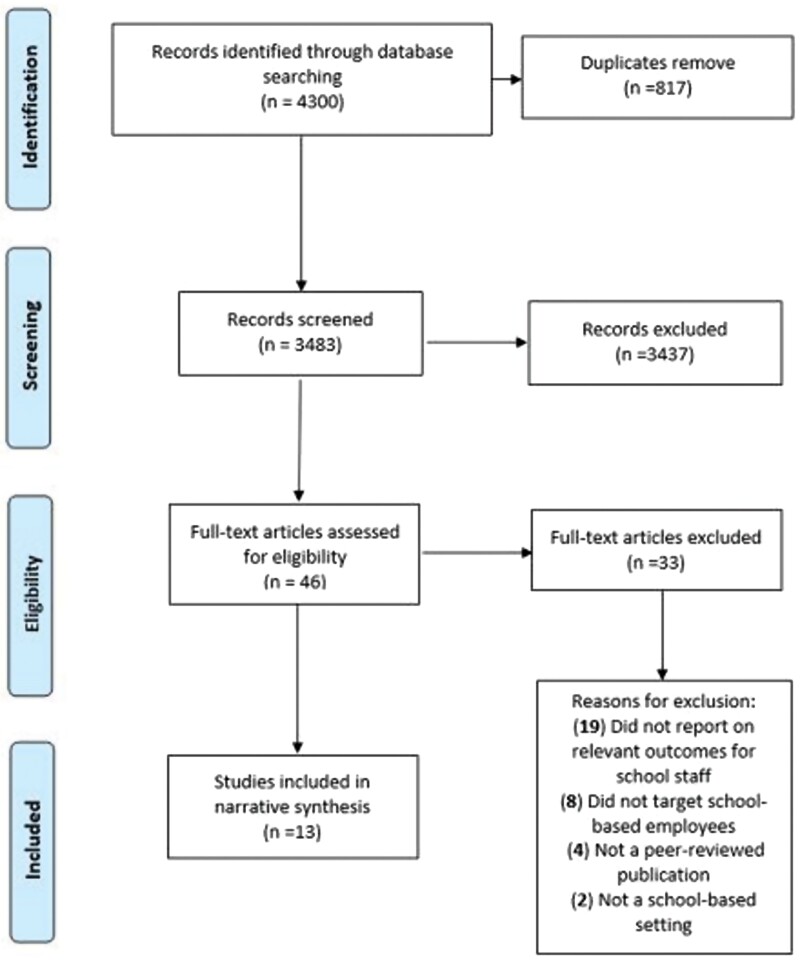
PRISMA flow diagram of included studies.

The searches retrieved 4300 relevant abstracts in total and after the removal of duplicates (*n* = 817), 3483 studies were screened by title and abstract as summarized in the PRISMA diagram (Figure 1). The full texts of 46 studies were reviewed and 13 papers were identified that met all inclusion criteria (Shi-Chang *et al.*, 2004; Cheung *et al.*, 2008; Chen *et al.*, 2010; Farag *et al.*, 2010; Siegel *et al.*, 2010; Berger-Jenkins *et al.*, 2014; Lemon *et al.*, 2014; Merrill and Sloan, 2014; Wang *et al.*, 2015; Frerichs *et al.*, 2016; Wang *et al.*, 2016; LeCheminant *et al.*, 2017; Kupolati *et al.*, 2019). Reasons for exclusion at the full text stage included no data reported on changes in staff behaviours, not an intervention study design and not a peer-reviewed publication (e.g. conference abstracts). One relevant abstract was identified via reference searching; however the full manuscript was not available in English and was therefore excluded.

**Fig. 2:**
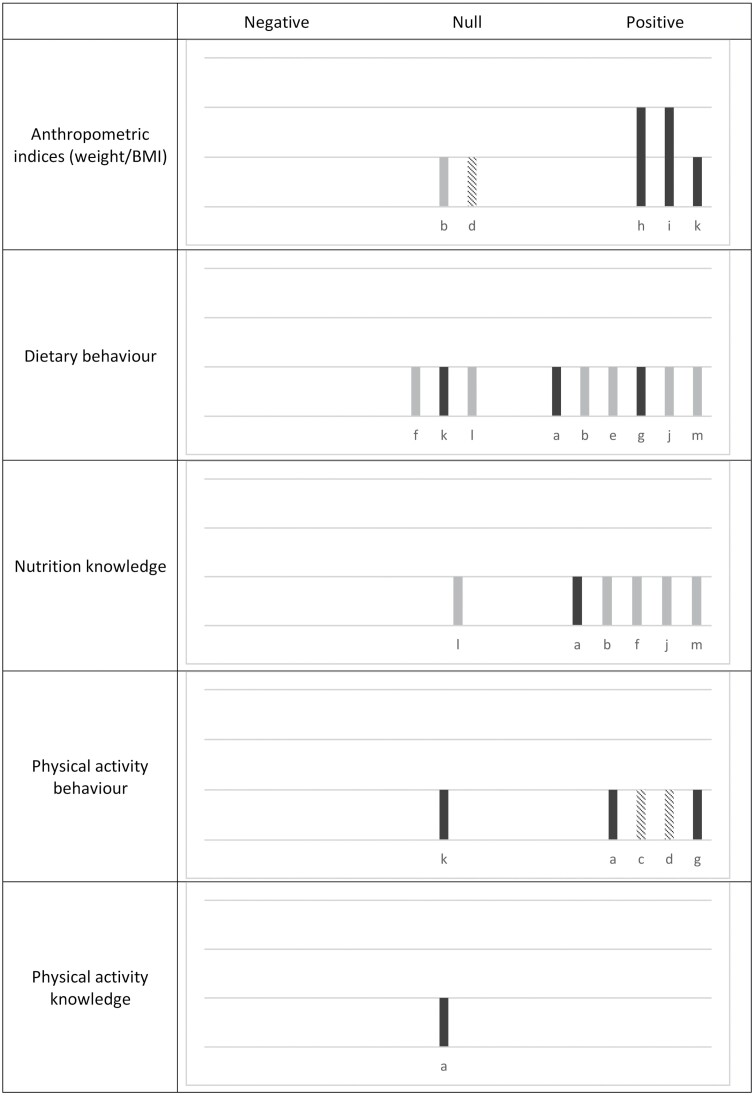
Harvest plot of the intervention effect of included studies reporting key obesity-related outcomes. Height depicts quality assessment [tall (third line) = good, medium (second line) = fair, low (first line) = poor]. Shading of bar = intervention focus (dark grey = physical activity and nutrition, light grey = nutrition, pattern = physical activity). Alphabet characters represent the studies. Studies: a (Berger-Jenkins *et al.*, 2014), b (Chen *et al.*, 2010), c (Cheung *et al.*, 2008), d (Farag *et al.*, 2010), e (Frerichs *et al.*, 2016), f (Kupolati *et al.*, 2019), g (LeCheminant *et al.*, 2017), h (Lemon *et al.*, 2014), i (Merrill and Sloan, 2014), j (Shi-Chang *et al.*, 2004), k (Siegel *et al.*, 2010), l (Wang *et al.*, 2016), m (Wang *et al.*, 2015).

The key characteristics of included studies are summarized in Table 1, and the detail of all extracted data is provided in Supplementary Table 2. Figure 2 provides a graphical summary of the results with a harvest plot of the intervention effect of studies reporting key obesity-related outcomes (anthropometric indices, dietary behaviour, nutrition knowledge, physical activity behaviour and physical activity knowledge). Six studies focussed specifically on increasing nutrition knowledge or behaviours, two on physical activity alone and five described interventions that addressed both physical activity and nutrition. Study designs included cluster-randomized control trials (four studies), quasi-experimental (four studies), and pre- and post-evaluations without a control group (five studies). Six studies included small sample sizes of participants (<50) (Cheung *et al.*, 2008; Farag *et al.*, 2010; Berger-Jenkins *et al.*, 2014; Frerichs *et al.*, 2016; Wang *et al.*, 2016; Kupolati *et al.*, 2019).

**Table 1:** Summary of included studies

Author, year of publication	Country	Design	Intervention focus	Strategy	Outcomes
Berger-Jenkins *et al.*, 2014	USA	Quasi-experimental	Physical activity + nutrition	School-wide marketing campaign, education, and coaching provided to staff	↑ nutrition knowledge scores^*^ ↑ diet scores^*^ ↑ physical activity behaviours
Chen *et al.*, 2010	Taiwan	Quasi-experimental	Nutrition	HPS Program implementation (whole of school approach) with nutrition focus	↑ nutrition knowledge^*^ Improved dietary intake behaviour
Cheung *et al.*, 2008	Hong Kong	Quasi-experimental	Physical activity	Marketing campaign (SMS, leaflets, posters)	↑ steps at work^*^
Farag *et al.*, 2010	USA	Pre- and post-intervention	Physical activity	Worksite wellness intervention (marketing, provision of equipment, encouragement of physical activity in planning periods).	↑ self-reported physical activity levels
Frerichs *et al.*, 2016	USA	Pre- and post-intervention	Nutrition	Building renovation followed strategies from the Healthy Eating Design Guidelines for School Architecture	↓ percentage of teachers with a high-fat diet^*^
Kupolati *et al.*, 2019	South Africa	Quasi-experimental	Nutrition	Treatment schools implemented a contextual nutrition education programme (NEP)	↑ nutrition knowledge scores
LeCheminant *et al.*, 2017	USA	Pre- and post-intervention	Physical activity + nutrition	Multi-component worksite wellness programme	↑ in days/week exercised^*^ ↑ min/week exercised^*^ ↑ fruit consumption^*^ ↑ vegetable consumption^*^
Merrill and Sloan, 2014	USA	Pre- and post-intervention	Physical activity + nutrition	Wellsteps wellness programme (behaviour change campaigns, incentives, biometric screening)	↓ BMI
Lemon *et al.*, 2014	USA	Cluster randomized controlled trial	Physical activity + nutrition	Intervention targeted environment, policy, organizational culture and individual knowledge, attitudes and skills	↓ weight ↓ BMI^*^
Shi-Chang *et al.*, 2004	China	Pre- and post-intervention	Nutrition	School-based working groups, nutrition training and resources for staff + school-wide health promotion initiatives	↑ nutrition knowledge^*^ ↑ attention to nutrition
Siegel *et al.*, 2010	USA	Cluster randomized controlled trial	Physical activity + nutrition	School tailored own health promotion interventions targeting staff nutrition, physical activity and stress	↓ BMI^*^
Wang *et al.*, 2016	China	Cluster randomized controlled trial	Nutrition	Formation of school nutrition group, training for teachers, posters, resources, peer support activities	No change in teachers’ nutrition-related knowledge, behaviour or attitudes
Wang *et al.*, 2015	China	Cluster randomized controlled trial	Nutrition	HPS intervention. Activities incl. formation of school nutrition group, training for teachers, posters, resources, peer support activities	↑ nutrition knowledge^*^ ↑ eating behaviour scores

Significant change.

Studies identified were predominantly in the USA (seven studies) and China (four studies), and there was one study each in South Africa and Taiwan. Four studies were implemented in schools located in rural areas (Farag *et al.*, 2010; Wang *et al.*, 2015; Frerichs *et al.*, 2016; Wang *et al.*, 2016).

Twelve of the 13 studies demonstrated positive impacts on either nutrition knowledge (Shi-Chang *et al.*, 2004; Chen *et al.*, 2010; Berger-Jenkins *et al.*, 2014; Wang *et al.*, 2015; Kupolati *et al.*, 2019), dietary behaviours (Chen *et al.*, 2010; Berger-Jenkins *et al.*, 2014; Frerichs *et al.*, 2016; LeCheminant *et al.*, 2017), BMI (Siegel *et al.*, 2010; Lemon *et al.*, 2014; Merrill and Sloan, 2014) or physical activity behaviours (Cheung *et al.*, 2008; Farag *et al.*, 2010; Berger-Jenkins *et al.*, 2014). One study showed no impact of the intervention on staff knowledge or behaviours (Wang *et al.*, 2016).

### Nutrition interventions

Six studies (Shi-Chang *et al.*, 2004; Chen *et al.*, 2010; Wang *et al.*, 2015; Frerichs *et al.*, 2016; Wang *et al.*, 2016; Kupolati *et al.*, 2019) focussed on improving the nutrition knowledge and behaviours of school staff. Chen *et al.* (Chen *et al.*, 2010) attempted to assess the impact of a government-initiated Health Promoting School (HPS) programme in Taiwan on staff nutrition knowledge and dietary behaviours. The quasi-experimental study included three study arms: HPS aimed at dietary intervention (*n* = 1), HPS not aimed at dietary intervention (*n* = 2) and non-health-promoting school (*n* = 2). Although no significant difference in BMI was found between the three study arms post-intervention, staff at HPS with a dietary intervention had significantly higher nutrition knowledge scores (*p* ≤ 0.001) than HPS not aimed at dietary intervention and non-health-promoting schools. Staff at HPS with a dietary intervention also reported better nutrient intake behaviours post-intervention such as eating breakfast, consuming five serves of vegetables and fruits and reading food labels (Chen *et al.*, 2010). However, without baseline measurements, and given the relatively small sample of schools involved, it is difficult to preclude pre-existing differences in behaviours.

Frerichs *et al.* (Frerichs *et al.*, 2016) studied the impact of environmental changes resulting from a school building renovation in the USA, guided by the evidence-based Healthy Eating Design Guidelines for School Architecture, on school staff members’ eating behaviours. The intervention included environmental changes such as the addition of a teaching kitchen, school garden, removal of vending machines and signage promoting healthy eating. The intervention was associated with a significant reduction in the proportion of school staff reporting consuming a high-fat diet [from 73.7 to 57.1% (*p* = 0.05)], and several staff health and wellness activities were initiated. Key weaknesses of this study included the small sample size and high attrition rate with greater than 50% lost to follow-up.

A pilot study, by Shi-Chang *et al.* (Shi-Chang *et al.*, 2004), also implemented a multi-level intervention (The China/WHO project), targeting all levels of the school from individual knowledge, to policy and infrastructure. The pre- to post-intervention design included approximately 700 employees in 12 schools (6 intervention and 6 control) and demonstrated an increase in nutrition knowledge from baseline to study end in the intervention schools. An improvement in nutrition behaviours of staff in intervention schools was also reported, with those staff self-reporting they were more likely to pay attention to the nutritional content of their lunch after the programme was implemented [an increase from 24 to 38% (*p* ≤ 0.01)]. While the proportion of staff reporting the same dropped in control schools. However, the measures of nutrition knowledge and behaviour used in the study were not robust, overall compromising the reliability of evidence of actual behaviour change or health outcomes. In addition, it was unclear if the staff surveyed at baseline and follow-up were in fact the same staff, further compromising the reliability of the evaluation of nutrition-related knowledge and behaviour change.

One small study by Wang *et al.* (Wang *et al.*, 2016) (*n* = 40 teachers surveyed) implemented a randomized intervention trial of a holistic school-based programme to improve nutrition knowledge, attitudes and behaviours in China. The study found no significant difference between intervention and control schools in staff members’ nutrition knowledge, attitudes and behaviours post-intervention (Wang *et al.*, 2016). A similar study, also conducted in China in 2015 (*n* = 60) aimed to improve nutrition knowledge and eating behaviours of students, parents and school staff by randomly assigning schools to either (i) a holistic intervention using the HPS framework, (ii) a partial intervention with a modified Health Education curriculum or (iii) a non-intervention control. School staff members’ nutrition knowledge increased over the course of the study for all three schools, with the largest improvements in nutrition knowledge among school staff in the partial intervention school (Wang *et al.*, 2015). Eating behaviour scores for staff also improved for all schools, with the greatest improvement seen in the school assigned to the HPS intervention. These studies were limited by their small sample size and short intervention duration (6 and 3 months, respectively).

Kupolati *et al.* (Kupolati *et al.*, 2019) used a quasi-experimental design to evaluate the implementation of a contextual nutrition education programme (comprising a teachers’ manual, picture book, learners’ workbooks and posters) in primary schools in Gauteng Province, South Africa. Pre- and post-testing were used in the small sample (12 teachers in the intervention school, 11 teachers in the comparison school) and showed significant improvement in intervention teachers’ nutrition knowledge post-implementation compared with controls. Improvements were also seen in dietary practices and nutrition attitudes; however these were not significant when compared with controls.

### Physical activity interventions

Two studies (Cheung *et al.*, 2008; Farag *et al.*, 2010) evaluated interventions targeting improvements in physical activity behaviours and knowledge. Cheung *et al.* (Cheung *et al.*, 2008) reported on a 6-week physical activity intervention in Hong Kong that aimed to increase the physical activity levels of school staff during school hours (*n* = 38 in the intervention group and 14 in the control). The intervention included text message reminders, education in the form of flyers and posters, as well as providing participants with pedometers (Cheung *et al.*, 2008). Following the intervention, the intervention group had a greater increase in steps at work compared with the control group (*p* < 0.001) (Cheung *et al.*, 2008). The study design did not allow for differentiation between the effectiveness of each element of the intervention, but a post-intervention survey found that pedometers were most commonly cited as motivating teachers in the intervention group to undertake more steps in their work day (Cheung *et al.*, 2008).

Farag *et al.* (Farag *et al.*, 2010) describe an intervention to promote physical activity among US school staff that included changing school environments to include physical activity equipment and promotion posters, and provision of pedometers and a physical activity handbook to individual staff members. No significant changes were seen pre- and post-intervention in self-reported physical activity levels [expressed in Metabolic Equivalent Task (MET) minutes per week, from 2337 [95% confidence interval (CI): 1521–3152] MET-minutes/week, to 2566 (95% CI: 1822–3309) MET-minutes/week] (Farag *et al.*, 2010).

### Interventions targeting both nutrition and physical activity

Five studies included both nutrition and physical activity outcomes (Siegel *et al.*, 2010; Berger-Jenkins *et al.*, 2014; Lemon *et al.*, 2014; Merrill and Sloan, 2014; LeCheminant *et al.*, 2017). Berger-Jenkins *et al.* (Berger-Jenkins *et al.*, 2014) describe a 2-year school-based intervention study named ‘Choosing Healthy and Active Lifestyles for Kids’ (CHALK) in the USA, which included a school-wide social marketing campaign. The intervention outcomes included changes in nutrition and physical activity knowledge, attitudes and behaviours of students and staff over time, with a control group added in year two of the study (Berger-Jenkins *et al.*, 2014). The intervention group showed significant improvements in staff nutrition knowledge (*p* ≤ 0.0001) and nutrition behaviour (*p* ≤ 0.0001) post-intervention. Self-reported physical activity also increased over time, however not to the point of statistical significance (*p* = 0.06). Post-intervention no significant differences were detected between the intervention and control group of school staff for nutrition and physical activity knowledge, attitude and behaviour in year 2 of the study. The evaluation had low response rates and high levels of attrition; of the 370 intervention group staff surveyed at baseline, only 99 completed follow-up surveys after 2 years, while among the control group the final survey included only 17 staff.

LeCheminant *et al.* (LeCheminant *et al.*, 2017) reported on a worksite wellness programme that included 1873 US school employees (75% female). The comprehensive outcome assessment included physical activity behaviours, fruit and vegetable consumption, restful sleep, smoking, alcohol consumption, self-rated health, mental health-related outcomes (stress, depression, life-satisfaction and loneliness) and job-related outcomes (job performance, absenteeism, job-related satisfaction). At the end of 2 years, participants reported a significant increase in physical activity with a 4.8% increase in days participating in exercise each week and a 12.8% increase in minutes participating in exercise each week compared with baseline. Dietary behaviour also improved with a 6.7% increase in fruit consumption (serves per day) and 4.1% increase in vegetable consumption (serves per day) (LeCheminant *et al.*, 2017). The study design did not include a control group and researchers did not measure how much (if at all) the employees engaged with the wellness programme over time, making it difficult to attribute the reported behaviour changes to the programme activities.

Two RCTs evaluated the impact of multi-component interventions in the USA. The study by Lemon *et al.* evaluated the impact of interventions targeting individual knowledge, attitudes skills, along with organizational culture and nutrition and physical activity policies among 782 school employees, across 12 public schools (Lemon *et al.*, 2014). At the end of the 2-year follow-up, there was a drop-out of 26.5% participants in the intervention group and 20.3% from the control group. The intervention group lost significantly more weight on average, relative to the control group (−1.37 kg, *p* = 0.04, equal to a reduction of 0.48 units in BMI). There were also improvements in nutrition knowledge and skills among staff who participated in the intervention. Another RCT, by Siegel *et al.* recruited 413 school staff from eight intervention and eight control schools (Siegel *et al.*, 2010). Employees in the intervention schools reduced their BMI by an average of 0.04 kg/m^2^, compared with the control group who increased their BMI by an average of 0.37 kg/m^2^, although overall sample sizes were small (Siegel *et al.*, 2010).

Merrill *et al.* undertook a large evaluation of the effectiveness of a worksite wellness programme in reducing health risk involving 2411 school staff in the USA (Merrill and Sloan, 2014). The intervention comprised nutrition education and physical activity initiatives in the workplace including biometric screening, culture change and behaviour change campaigns. Post-intervention, 46.0% of all participants in this pre- and post-test study design reduced their BMI, along with reductions in blood pressure, blood cholesterol and blood glucose (Merrill and Sloan, 2014).

### Quality assessment

Overall, the studies included were assessed to be of moderate to high risk of bias. No quality threshold was applied, and therefore the results of all 13 included studies is reported here. Most studies had small samples sizes and did not include a control group to assess the effectiveness of the interventions (see Supplementary Table 3). Five of the included studies did not clearly describe their inclusion criteria and four studies did not clearly describe the interventions. Out of the total 13 studies, four did not describe an intervention that would have been equally distributed across the study population (e.g. some parts of the intervention may not have reached all school staff, with teachers being more likely to be exposed).

## DISCUSSION

The findings of this review demonstrate a lack of research focus and investment in interventions targeting school staff and teachers’ health over the past two decades internationally. Despite widespread acknowledgement of the importance of school staff as an important population for health promotion, there have only been 13 studies published since 2000, and the majority used relatively weak study designs and included small numbers of schools and staff members. In this review, we found that school staff focussed health promotion interventions positively impacted dietary intake, weight or BMI or physical activity outcomes for staff.

The lack of studies in this review is especially striking when compared with school-based obesity prevention interventions for children, of which there have been at least 50 high quality randomized—or cluster randomized—controlled trials published over the same period, largely in the USA, UK and Europe (Liu *et al.*, 2019). A recent systematic review and meta-analysis of the effect of school-based obesity prevention interventions on BMI or BMI-*z* score of children and found significant reductions in intervention schools compared with controls for both single- and multi-component interventions (Liu *et al.*, 2019). Such school-based studies have the potential to also impact the staff they employ, however the direct impacts remain unknown because interventions and evaluations rarely focus on school staff.

Despite good evidence internationally for workplace health promotion, we found that in the school setting high quality studies evaluating the impacts on staff are lacking. The staff focussed interventions identified in this review showed promise, especially those that aimed to improve both physical activity and diet, however sample sizes were small and research designs lacked robustness, severely limiting the generalizability of results. Almost half of the included papers had an evaluation sample comprising fewer than 50 participants, and despite comprehensive intervention strategies, were under-powered to identify intervention effects. Also, the main positive findings were of knowledge gain, which we know is a very early step in the complex pathway to behaviour change.

Several of the studies that used multi-level interventions showed promise (Shi-Chang *et al.*, 2004; Chen *et al.*, 2010; Farag *et al.*, 2010; Lemon *et al.*, 2014; Merrill and Sloan, 2014; Wang *et al.*, 2015; LeCheminant *et al.*, 2017). Addressing multiple drivers of obesity and ill-health through more comprehensive approaches recognizes the complex nature of behaviour change and preventing obesity; however, far more research would be required to identify and prioritize the most effective strategies and approaches to achieve healthier behaviours and weight status among school staff.

Schools have been identified as a priority target for addressing childhood overweight and obesity (Dehghan *et al.*, 2005). An important consideration for research focussed on school staff health and behaviour, is that staff are also in a position to facilitate improvements in children’s health behaviours, and subsequently overweight and obesity rates. A recent study showed that teachers who are focussed on improvements in their own health tend to create classrooms in which obesity prevention efforts are better supported (Esquivel *et al.*, 2016). For example, a teacher who is actively increasing their physical activity level, along with experiencing positive health effects of the change, will be more likely to encourage physical activity among their students (Esquivel *et al.*, 2016). Joint interventions, with components tailored towards improving both staff and students’ health have the potential to prevent obesity across the lifespan, in both children and adults, and should be priority areas for future research.

It is recognized that the working conditions of the school-based workforce can be stressful, characterized by high job stress-related workers compensation claims, high burnout reflecting high workload requirements and poor work/life balance (Lever *et al.*, 2017; Arvidsson *et al.*, 2019). As outlined in the WHO Healthy Workplaces Model, interventions targeting the school-based workforce should address both the working conditions that affect health behaviours and offering health behaviour change to optimize impacts (World Health Organisation, 2007). This is an important consideration for future research.

### Strengths and weaknesses

This is the first systematic review of the literature to synthesize the evidence on interventions targeting the health of school staff internationally. A strength of this study is that we used broad search terms in five databases, across literature from the years 2000 to 2020. Despite this, there was only a small number of highly diverse, and largely poor quality, studies that met the inclusion criteria, and this precluded a meta-analysis. As with all systematic reviews, the evidence synthesis here could be limited by publication bias, where studies with neutral or negative results may not be published thus skewing results.

## CONCLUSIONS

This review demonstrates extensive gaps in the evidence base for interventions that seek to improve the health of teachers and school staff, globally. Studies are needed across all contexts and especially in rural or disadvantaged communities. Although the interventions here showed promise in improving diet and physical activity in school staff and associated health risk factors, the health of this large and influential population group needs further focus and research investment.

## Supplementary Material

Supplementary material is available at *Health Promotion International* online.

Supplementary Table 1: Search strategy terms.

Supplementary Table 2: Detailed data extraction.

Supplementary Table 3: Quality assessment of included studies.

daac061_suppl_Supplementary_Table_S1

daac061_suppl_Supplementary_Table_S2

daac061_suppl_Supplementary_Table_S3

## Protocol and Registration

This systematic review was conducted and reported following the Preferred Reporting Items for Systematic Reviews and Meta-Analysis (PRISMA) statement guidelines (Moher *et al.*, 2009) and followed the pre-specified methods documented in the review protocol as submitted to the International Prospective Register of Systematic Reviews (PROSPERO ID: CRD42019127773) on 14 March 2019.
