# Supplementary material for: Systematic review of the effectiveness of health promotion interventions targeting obesity prevention in school-based staff
Source: Health Promot Int. 2022 Jul 5;37(3):daac061. doi: 10.1093/heapro/daac061 (PMC9437818; doi:10.1093/heapro/daac061)
Supplement: daac061_suppl_Supplementary_Table_S1 [file daac061_suppl_supplementary_table_s1.docx]

Supplementary Table 1: Search Strategy Terms

| Basic search strategy: | 1. School* 2. Staff* OR teacher* OR employee* OR worker* OR workplace 3. Intervention* OR program* OR “health promotion” OR prevent* OR strateg* OR initiative* 4. Diet* OR nutrition* OR eating OR consumption OR weight OR overweight OR obes* OR “physical* activ*” OR “sedentary behavio*” OR exercise 5. AND 2 AND 3 AND 4   Limits: date of publication 2000 -2019, English language, Academic Journals |
| --- | --- |
| Search strategy (Medline): | 1. AB exercise OR TI exercise OR AB "physical* activ*" OR TI "physical* activ*" OR AB "sedentary behavio*" OR TI "sedentary behavio*" OR AB diet* OR TI diet* OR AB nutrition* OR TI nutrition* OR AB eating OR TI eating OR AB weight OR TI weight OR AB overweight OR TI overweight OR AB obes* OR TI obes* 2. (MH "Health Education") OR (MH "Health Promotion") OR (MH "Weight Reduction Programs") OR (MH "Healthy People Programs") OR AB intervention* OR TI intervention* OR AB program* OR TI program* OR AB prevent* OR TI prevent* OR AB strateg* OR TI strateg* OR AB initiative* OR TI initiative* 3. AB school* OR TI school* OR (MH "Schools") 4. AB staff* OR TI staff* OR AB employee* OR TI employee* OR AB worker* OR TI worker* OR AB workplace OR TI workplace OR AB teacher* OR TI teacher* OR (MH "School Teachers") 5. AND 2 AND 3 AND 4   Limits: date of publication 2000 -2019, English language, Academic Journals |
