# Supplementary material for: Systematic review of the effectiveness of health promotion interventions targeting obesity prevention in school-based staff
Source: Health Promot Int. 2022 Jul 5;37(3):daac061. doi: 10.1093/heapro/daac061 (PMC9437818; doi:10.1093/heapro/daac061)
Supplement: daac061_suppl_Supplementary_Table_S2 [file daac061_suppl_supplementary_table_s2.docx]

Supplementary Table 2: Detailed Data Extraction

| Author, year of publication | Year(s) of study | Setting/Study design/Country | Intervention strategies | Participants, follow up | Outcome measures | Results | Conclusions |
| --- | --- | --- | --- | --- | --- | --- | --- |
| **Berger-Jenkins et al, 2014**  *Evaluation of a coordinated school-based obesity prevention program in a Hispanic community: choosing healthy and active lifestyles for kids/healthy schools healthy families* | 2008-2010 | Elementary (primary) public schools.  Primarily Hispanic community in New York, USA  Longitudinal study design. | Comprehensive obesity prevention program: Choosing Healthy & Active Lifestyles for Kids (CHALK)  Main strategy: school-wide social marketing campaign.  Obesity prevention program – 2 years. Staff received 1) Staff development series and workshops on healthy nutrition and physical activity;  2) school wellness coach (governing body – met monthly to discuss & plan health goals for the school); 3) healthy habits newsletter – monthly. School-wide nutrition fairs were held twice a year. | N=99 school staff (teachers, administrators, and ancillary staff e.g. school aides and cafeteria personnel) were included in evaluation analysis for entire 2 year period (completed all 4 surveys).  Extra 64 staff were included in 2^nd^ year analysis (completed both year 2 surveys) | Self-reported staff survey relating to healthy nutrition and PA assessing:  -knowledge  -attitude  -behaviour | *Knowledge:*  Nutrition knowledge score: ↑ (P = <.0001)  PA knowledge score: no sig change (P = .35)  *Attitude:*  Nutrition attitude: high at the onset of the study and didn’t change sig over time  Self efficacy to exercise ↑ (P = .004)  No sig differences between intervention and control in changes in nutrition or PA attitudes in year 2.  *Behaviour:* intervention staff reported a healthier diet over time (P < .0001)  No sig diff in change in nutrition behaviour b/w intervention and control in yr 2  PA behaviour improved (P = .06)  No sig diff in change in PA behaviours b/w intervention and control in yr 2 | Comprehensive school-based obesity prevention programs that utilise a public health approach and target students, adults, and communities may be effective at improving nutrition and physical activity knowledge, attitudes and behaviour in large, under-resourced public elementary schools in underserved Hispanic communities. |
| **Chen et al** (2009)  *Significant effects of implementation of health-promoting schools on school teachers’ nutrition knowledge and dietary intake in Taiwan* | 2007 | 4 elementary schools in Taiwan – 2 intervention (HPS) and 2 controls  Non-randomised, controlled study. | HPS program initiated in Taiwan in 2003 by the Dept of Health and the Ministry of Education with 6 major elements in line with WHO guidelines. ‘Nutrition and diet’ is a special intervention programme in addition to the 6 major elements that schools can choose to adopt. | N=283  3 groups:  HP-D: HPS aiming at dietary intervention  HP-ND: HPS not aiming at diet intervention  NHP: non HP schools | Self-reported questionnaire assessing  -nutrition knowledge score  - dietary intake  - ht  -wt  -BMI  -wt perception | -Nutrition knowledge score significantly higher in the HP-D group (P<.0.001)  -better dietary intake behaviours (HP-D group)   - eating BF - consuming 5 serves of veg and fruit daily - reading food labels for nutrient content   - BMI: no significant change | Implementation of a coordinated health promoting schools framework on nutrition and diet positively correlated with school teachers’ nutrition knowledge and dietary intake. |
| **Cheung et al** (2008)  *Using environmental stimuli in physical activity intervention for school teachers: a pilot study* | Not listed | Teachers at Primary schools in Hong Kong.  Non-randomised control trial | PA intervention aiming to promote teachers’ PA level during work hours: SMS messages, leaflets, posters promoting walking, pedometer. | N=38 teachers from 3 intervention schools  14 teachers from 1 control school | -pedometer readings of steps taken  -Stage of Change scores  recorded before and after intervention | -Steps at work ↑ (P<0.001)  -The ANCOVA for stage of change for status groups was non-significant | An intervention utilising environmental stimuli as the strategy can be successfully applied in the school setting for the promotion of school teachers’ PA |
| **Farag et al (2010)**  *Evaluation of a community-based participatory physical activity promotion project: effect on cardiovascular disease risk profiles of school employees* | 2005-2006 | Rural public school system in Southwestern Oklahoma, USA  Pre and post intervention design. | Focus of the intervention was on promoting physical activity. Opportunities for in school physical activity were created by marking hallways, adding a treadmill in each school, and allowing teachers to use planning periods for physical activity. | 5 schools involved  N=187  Post-intervention CVD screening completed at 6 months | body composition, blood pressure, lipids, glucose and self-reported physical activity levels (IPAQ-short 7) | Self-reported physical activity levels ↑ (Sign t = -1.901, p = 0.06), however not statistically significant  BMI did not change  Reduced cholesterol and BP | A successful participatory program was associated with improvements in several CVD risk factors among school employees. |
| **Frerichs et al (2016)**  *The Role of school design in shaping healthy eating-related attitudes, practices, and behaviours among school staff* | 2012 | 3 elementary schools in a rural county school district in Virginia, USA consolidated and integrated into one fully renovated school building in 2012  Pre and post intervention design | The building renovation followed strategies from the Healthy Eating Design Guidelines for School Architecture incl:  -removal of vending machines  -signs promoting healthy eating  -providing a teaching kitchen  -displaying healthy cafeteria items | N=41 staff completed pre and post occupancy surveys.  Surveys, structured interviews, and semistructured in-depth interviews were conducted prior to and at 12 months post-occupancy in the renovated school building. | Food frequency questionnaire to est fat, F & V intake | Sig decrease in the percent of teachers with a high-fat diet (from 73.68% to 57.14%, p < .05)  Fruit and vegetable intake did not significantly change.  The school implemented new policies and programs, including staff wellness activities. | The findings reveal promise that changes to the physical environment of a school can facilitate and support adoption of healthy eating policies and practices. The school in this study implemented new healthy eating policies and practices, and there was evidence that social interaction that placed value on healthy eating and active lifestyles emerged. |
| **Kupolati (2019)**  *A Contextual Nutrition Education Program Improves Nutrition Knowledge and Attitudes of South African Teachers and Learners* | 2015 | Schools in the Bronkhorstspruit district, Gauteng Province, South Africa participated in the study  Quasi-experimental study | 2 primary schools were randomly selected to implement a contextual nutrition education program (NEP). The treatment school teachers taught nutrition using a developed nutrition education manual, while the control school teachers taught nutrition in the usual manner. | 23 teachers who taught nutrition in Grades 4–7 (treatment school, n = 12) and 681 learners (treatment school, n = 350) participated in the study. | -total nutrition knowledge score  -dietary practices  -nutrition attitudes | Post-implementation, the treatment school teachers’ had higher total nutrition knowledge mean score (85.5% ± 8.2, p = 0.003) compared to the control school. Within the treatment school, total nutrition knowledge mean score of the teachers improved by 14.1%, p ≤ 0.001.  The dietary practices of the teachers and the learners, and the nutrition attitudes of the teachers in the treatment school showed no significant within school improvement or in comparison with the control school (p > 0.025). | The NEP led to the improvement in the teachers’ and the learners’ nutrition knowledge and the learners’ nutrition attitudes. However, no significant improvement in the dietary practices of either teachers or learners was found. |
| **LeCheminant (2017)**  *Changes in Behaviors and Outcomes Among School-Based Employees in a Wellness Program* |  | Employees of a large public school district in the western United States  Longitudinal | The wellness program included multiple components, such as administrative planning, culture evaluation and analysis, baseline health behavior and biometric evaluation, strategies to improve communication, and behaviour change campaigns | n=1873  The school district was comprised of 6 high schools, 8 junior high schools, and 31 elementary schools  baseline, 1-year, and 2-year follow-up results for the school employees enrolled in the comprehensive worksite wellness program | -exercise level -fruit and vegetable consumption -restful sleep -smoking  -alcohol consumption  -self-rated health  -mental health-related outcomes  -job-related outcomes | 4.8% ↑ in days/week exercised (p<.0001)  12.8% ↑ minutes/week exercised (p<.0001)  6.7% ↑ fruit consumption (p<.0001)  4.1% ↑ vegetable consumption (p<.0001)  ↑ days per week of restful sleep  ↓ alcohol consumption  ↓ smoking  Several mental health-related outcomes improved  job performance was slightly lower (↓ 2%), and the other job-related outcomes were unchanged | School employees may benefit from a comprehensive worksite wellness program, particularly to improve health behaviours. However, mental health and job-related outcome results were mixed over 2 years. |
| **Merrill & Sloan (2014)**  *Effectiveness of a health promotion program among employees in a western United States school district.* | 2011-12 | Employees from a school district in the western United States.  Pre and post intervention design | Wellsteps wellness program. The intervention incorporated administrative planning, baseline data evaluation, culture change and communication strategy analysis, biometric screening, and behavior change campaigns. | Analyses were based on 2411 employees that participated in the wellness program for 12 months. | -BMI  -blood pressure  -chol  -glucose | 46.0% ↓ body mass index, however mean BMI did not change.  The percentage of employees moving from the obese group to the overweight group was 11.1%, and from the overweight group to the normal weight group was 11.6% | The worksite wellness program effectively lowered risk measures among those identified in high-risk categories at baseline.  Individuals who had higher biometric readings at baseline were more likely to improve health status by the end of the intervention than individuals with normal baseline readings. |
| **Lemon et al (2014)** | 2009-2012 | Massachusetts public high schools.  Cluster randomized controlled trial | The intervention targeted the nutrition and  physical activity environment and policies, the organizational culture, and individual knowledge, attitudes and skills | 782 employees from 12 schools (baseline, 12 months and 24 months) | Primary outcome was change in BMI from baseline to 12 and 24 month follow-up. Change in weight as well as intervention implementation was measured | At 24-month follow-up, there was a mean change of −3.03 pounds  (p = .04) and of −0.48 BMI units (p = .05) between intervention and comparison conditions. The majority of  intervention strategies were implemented by all intervention schools | The study provides evidence that a multi-level intervention integrated within the organizational culture  Can lead to reductions in weight and BMI in school employees. |
| **Shi-Cheng et al** (2004) | 2000 | Zhejiang Province, China.  Pre and post intervention design. | Interventions related to school staff included  establishing school-based working groups, nutrition training and resources  for staff, along with  school-wide health promotion initiatives | Three primary and three secondary schools (n=800 teachers and school staff) (pre and post intervention follow up) | Nutrition knowledge, nutrition behaviours | Nutrition knowledge of school staff increase from baseline to the end of the intervention period. When taking lunch at school,  the percentage of staff who reported paying  attention to nutrition increased from 24 to 38%  (p 0.01) at pilot schools while it decreased  from 27 to 19% (p 0.01) at control schools | It is feasible to use nutrition training and school-wide health promotion activities to  improve the dietary knowledge, behaviour and attitude of school staff. |
| **Siegel at al**, (2010) | 2005-2007 | Schools in two geographic areas of Los Angeles,  California.  Randomized controlled trial | Schools formed committees and tailored their own health promotion interventions targeting staff from nutrition, physical activity and addressing stress | 16 elementary schools, 8 controls (n=143 staff) and 8 intervention (n=145 staff) | -BMI  -Waist-hip ratio  -Physical activity (mins)  Fruit and vegetable consumption | Employees  in intervention schools reduced their BMI by an average of 0.04 kg/m2  , and those  in control schools increased their BMI by an average of 0.37 kg/m2  . Comparisons  for waist–hip ratio, weekly physical activity minutes, and fruit and vegetable  Consumption were not significant. | Interventions using a co-design approach are effective at stimulating organisational change and to prevent weight gain in school staff. |
| **Wang et al** (2016)  *A Holistic School-Based Nutrition Program Fails to Improve Teachers' Nutrition-Related Knowledge, Attitudes and Behaviour in Rural China* | 2012-2013 | Beijing China  Cluster randomised controlled trial | Holistic health promoting schools intervention | Two schools, one was the control (n=20) and the other was the intervention (n=20) | -Nutrition-related knowledge, -attitudes  and behaviour ( pre- and post-intervention) | There was no change between the two groups. | This study found that the intervention had no effect on teachers nutrition related knowledge, behaviour or attitudes. |
| **Wang et al** (2015)  *Do health-promoting schools improve nutrition in China?* | 2015 | Sample: Chinese rural middle school students, parents and school staff. Country: China.  Cluster randomised controlled trial | Core information component of interventions of HPS and HE intervention contained the definition and importance of a balanced diet, the functions of the nutrients, nutrient deficiencies and their effects, how to supplement necessary nutrients reasonably, good hygienic practices and food safety. | Three schools  1. Control school  2. Holistic intervention school using the health-promoting school (HPS) framework  3. Partial intervention school with modified Health Education curriculum (HE)  There were 20 staff in each school who completed the survey (n=60) | -Nutrition knowledge  - eating behaviours were measured at baseline and 3-month after interventions | ↑nutrition knowledge* eating behaviours: largest increase among school staff (0.83 point). Largest increase observed in the HPS school (from 3.45 to 4.45). | Schools with the HPS intervention had the greatest benefit on staff nutrition knowledge and eating behaviours compared to the health education only and control schools |
