# Supplementary material for: Systematic review of the effectiveness of health promotion interventions targeting obesity prevention in school-based staff
Source: Health Promot Int. 2022 Jul 5;37(3):daac061. doi: 10.1093/heapro/daac061 (PMC9437818; doi:10.1093/heapro/daac061)
Supplement: daac061_suppl_Supplementary_Table_S3 [file daac061_suppl_supplementary_table_s3.docx]

Supplementary Table 3: Quality Assessment

|  | Berger-Jenkins et al (2014) | Chen et al (2009) | Cheung et al (2008) | Farag et al (2010) | Frerichs et al (2016) | Kupolati et al (2019) | LeCheminant (2017) | Lemon et al (2014) | Merrill & Sloan (2014) | Shi-Cheng et al (2004) | Siegel at al (2010) | Wang et al (2016) | Wang et al (2015) |
| --- | --- | --- | --- | --- | --- | --- | --- | --- | --- | --- | --- | --- | --- |
| Was the study question or objective clearly stated? | Y | Y | Y | N | Y | Y | Y | Y | Y | N | Y | Y | Y |
| Were eligibility/selection criteria for the study population prespecified and clearly described? | N | N | Y | Y | N | N | Y | Y | Y | Y | Y | Y | Y |
| Were the participants in the study representative of those who would be eligible for the test/service/intervention in the general or clinical population of interest? | Y | N | N | Y | N | Y | Y | Y | Y | Y | Y | N | Y |
| Were all eligible participants that met the prespecified entry criteria enrolled? | N | Y | N | Y | N | Y | N | Y | Y | Y | Y | N | Y |
| Was the sample size sufficiently large to provide confidence in the findings? | N | N | N | Y | N | N | Y | Y | Y | Y | Y | N | N |
| Was the test/service/intervention clearly described and delivered consistently across the study population? | N | N | Y | Y | N | Y | Y | Y | Y | Y | N | Y | Y |
| Were the outcome measures prespecified, clearly defined, valid, reliable, and assessed consistently across all study participants? | Y | Y | Y | Y | Y | Y | Y | Y | Y | Y | Y | Y | Y |
| Were the people assessing the outcomes blinded to the participants' exposures/interventions? | N | N | N | N | N | N | N | N | N | N | N | N | N |
| Was the loss to follow-up after baseline 20% or less? Were those lost to follow-up accounted for in the analysis? | N | NA | Y | Y | N | Y | N | N | N | N | N | Y | Y |
| Did the statistical methods examine changes in outcome measures from before to after the intervention? Were statistical tests done that provided p values for the pre-to-post changes? | Y | N | Y | Y | Y | Y | Y | Y | Y | N | Y | Y | Y |
| Were outcome measures of interest taken multiple times before the intervention and multiple times after the intervention (i.e., did they use an interrupted time-series design)? | N | N | N | N | N | N | N | N | N | N | N | N | N |
| If the intervention was conducted at a group level (e.g., a whole hospital, a community, etc.) did the statistical analysis take into account the use of individual-level data to determine effects at the group level? | Y | Y | NA | N | N | N | N | Y | Y | N | N | N | N |
| **QUALITY RATING (good/fair/poor)** | **poor** | **poor** | **poor** | **poor** | **poor** | **poor** | **poor** | **fair** | **fair** | **poor** | **poor** | **poor** | **poor** |

*accessed from <https://www.nhlbi.nih.gov/health-topics/study-quality-assessment-tools>.
